# The association between visceral adiposity index and decreased renal function: A population-based study

**DOI:** 10.3389/fnut.2023.1076301

**Published:** 2023-03-10

**Authors:** Zheng Qin, Xinyang Chen, Jiantong Sun, Luojia Jiang

**Affiliations:** ^1^West China School of Medicine, West China Hospital of Sichuan University, Chengdu, China; ^2^Department of Nephrology, Jiujiang No.1 People’s Hospital, Jiujiang, China

**Keywords:** visceral adiposity index, chronic kidney disease, albuminuria, estimated-glomerular filtration rate, cross-sectional study

## Abstract

**Aims:**

We aimed to investigate the association of visceral adiposity index (VAI) with decreased renal function in US adults.

**Design and methods:**

Cross-sectional data were analyzed for 35,018 adults in the National Health and Nutrition Examination Survey (NHANES) 2005–2018. VAI was determined using waist circumference, body mass index (BMI), triglycerides (TGs) and high-density lipoprotein-cholesterol. Albuminuria was defined as urinary albumin-to-creatinine ratio (ACR) >30 mg/g. A low estimated-glomerular filtration rate (eGFR) was defined as an eGFR lower than 60 ml/min/1.73 m^2^. Chronic kidney disease (CKD) was defined as either albuminuria or low-eGFR. A multivariable logistic regression analysis was utilized to explore the relationship of VAI with albuminuria, low-eGFR and CKD. Subgroup analysis and interaction tests were also conducted.

**Results:**

A total of 35,018 participants were enrolled with albuminuria, low-eGFR, and CKD prevalence rates of 5.18, 6.42, and 10.62%, respectively, which increased with the higher VAI tertiles. After full adjustment, a positive association of VAI with albuminuria (OR = 1.03, 95% CI: 1.00, 1.06) and CKD (OR = 1.04, 95% CI: 1.02, 1.06) was observed. Participants in the highest VAI tertile had a significantly 30% increased risk for albuminuria (OR = 1.30, 95% CI: 1.07, 1.58) and a 27% increased risk for CKD (OR = 1.27, 95% CI: 1.08, 1.49) compared with those in the lowest VAI tertile. No statistically significant association between VAI and low-eGFR was detected. Subgroup analysis and the interaction term indicated that there was no significant difference among different stratifications.

**Conclusion:**

Visceral adiposity accumulation evaluating by VAI was associated with increased likelihood of the decline in renal function.

## 1. Introduction

The damage of renal function could be reflected in albuminuria, a decrease in the estimated-glomerular filtration rate (eGFR) and even the development of chronic kidney disease (CKD) (1). Approximately 5–19% of the general population suffer from albuminuria, which is a putative marker of an impaired glomerular filtration barrier and abnormal urinary albumin excretion (2–4). Decreased renal function is usually defined as eGFR <60 mL/min/1.73 m^2^ (5). Both albuminuria and low-eGFR are not only markers of early kidney disease, but also predictors of CKD progression and cardiovascular disease (6, 7). CKD is a chronic condition presented as kidney structural or functional abnormalities caused by multiple factors with a global prevalence of 10.5–13.1%, which represents a significant global health burden as well (8, 9). Globally, the incidence of CKD increased by 89%, prevalence increased by 87%, death due to CKD increased by 98%, and disability-adjusted-life-years (DALYs) increased by 62% from 1990 to 2016 (10).

Visceral Adiposity index (VAI), which is also known as visceral fat grade, has proven to be a reliable indicator of visceral fat accumulation and dysfunction in adipose tissue (11, 12). It was calculated using anthropometric [waist circumference (13), body mass index (BMI)] and metabolic parameters [triglyceride (TG) and high density lipoprotein-cholesterol (HDL-C) concentrations] to evaluate visceral obesity function, which has been broadly used in previous studies (14). The higher the index value, the higher the content of visceral fat. Compared to other traditional body assessment parameters including BMI, WC, and waist-to-height ratio (WHtR), VAI can accurately distinguish visceral adiposity from subcutaneous adiposity. Magnetic Resonance Imaging (MRI) and Computed Tomography (CT) are precise and reliable to identify visceral fat, however, these machine-based measurements can be costly and difficult to conduct for some individuals. Thus, using VAI, a mathematical model that takes into account both anthropometric and metabolic parameters to evaluate the adipose distribution may be a better tool for assessing the impacts of visceral adiposity on clinical outcomes. Many studies have confirmed the positive predictive value of VAI for insulin insensitivity and diabetes (15–18). Moreover, VAI is significantly associated with cardiovascular diseases, such as hypertension and arterial atherosclerosis (19–22). The correlation of VAI with non-alcoholic fatty liver disease (NAFLD) (23, 24), metabolic syndrome (MS) (25), and some tumors (26, 27) has also been widely reported. More particularly, VAI may also be a superior predictor for kidney disease. Several studies have suggested a relationship between visceral fat and renal function. An epidemiological study in Netherlands revealed that visceral fat was associated with microalbuminuria in women while liver fat was not, which was supported by Mendelian randomization (28). Another retrospective study including 14,529 male and 10,561 female adults in China demonstrated independent predictive power of visceral obesity on renal damage in all ages and both genders, except men under 45 years of age (29). Moreover, there is evidence to suggest that obesity, especially central obesity, is an important risk factor for CKD (30–33). However, the association has not been studied in a nationally representative sample of US adults.

Hence, we investigated whether VAI was associated with decreased renal function among National Health and Nutrition Examination Survey (NHANES) subjects. Our hypothesis was that an elevated VAI would increase the risk of reduced kidney function.

## 2. Materials and methods

### 2.1. Survey description

National Health and Nutrition Examination Survey is a national population-based cross-sectional cohort study to assess the nutrition and health status of non-institutionalized civilian populations in the US (34). For recruitment of a representative sample of the US population, a stratified, multistage and probability sampling design was used.

The National Center for Health Statistics (NCHS) Research Ethics Review Board approved all NHANES protocols. All survey participants provided written informed consent. A detailed description of the NHANES study and its data is available online at https://www.cdc.gov/nchs/nhanes/.

### 2.2. Study population

We used the NHANES survey cycles 2005–2018 because these surveys have provided complete data to calculate the VAI, urinary albumin: creatinine ratio (ACR) and eGFR using the same protocols. An in-home interview and a physical examination were conducted at a mobile examination center to collect blood and urine samples.

**FIGURE 1:**
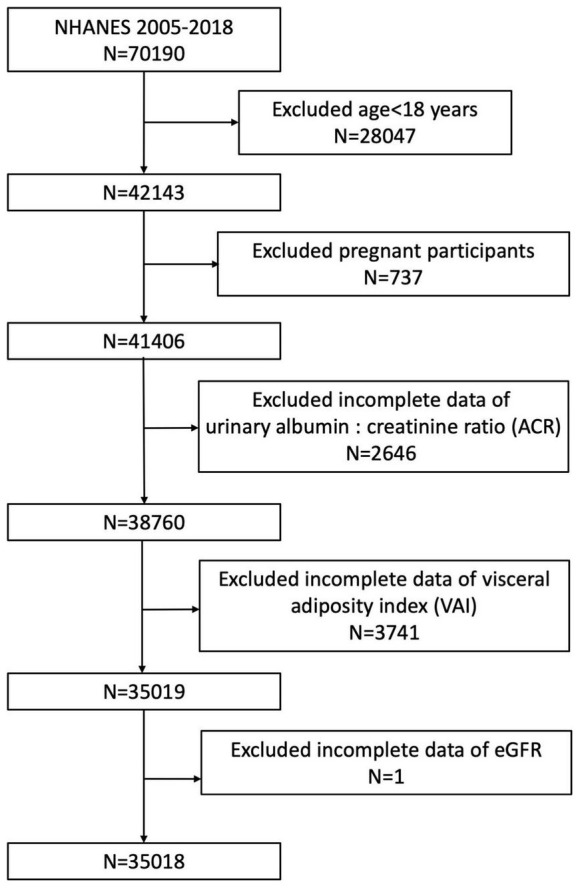
Flowchart of the sample selection from NHANES 2005–2018.

The analysis included participants with complete information about VAI and renal function. There were initially 70,190 participants enrolled in the study. After excluding participants aged <18 years (*n* = 28047), pregnant (*n* = 737), missing data about ACR (*n* = 2646), VAI (total, *n* = 3741; BMI, *n* = 445; TG, *n* = 2124; WC, *n* = 1163; HDL-C, *n* = 9), and eGFR (*n* = 1), our final analysis included 35,018 eligible participants (Figure 1).

### 2.3. Definition of visceral adiposity index, albuminuria, low-eGFR and chronic kidney disease

Visceral adiposity index is a gender-specific index that estimates visceral adiposity by combining WC, BMI, TG, and HDL-C. Higher VAI scores indicate more estimated visceral fat. The VAI for each participant was calculated by using the following formulas (19). For males: VAI = WC/(39.68 + (1.88 × BMI)) × (TG/1.03) × (1.31/HDL-C); For females: VAI = WC/(36.58 + (1.89 × BMI)) × (TG/0.81) × (1.52/HDL-C). TG and HDL-C were calculated in mmol/L, and WC was calculated in cm in the formulas. The VAI was analyzed as a continuous variable and further analysis was conducted by grouping participants according to their VAI tertiles.

A single, spot urine sample was used to determine urinary albumin and creatinine using a solid-phase fluorescent immunoassay and a modified Jaffe kinetic method. Based on the urinary ACR, we defined albuminuria as ACR >30 mg/g. Serum creatinine was measured using the Jaffe rate method and calibrated isotope dilution mass spectrometry. The CKD Epidemiology Collaboration (CKD-EPI) creatinine equation was used to calculate eGFR based on data about gender, race, age, and SCr (35). Low-eGFR was defined as an eGFR lower than 60 ml/min/1.73 m^2^. CKD is characterized by albuminuria or a low-eGFR as defined by Kidney Disease: Improving Global Outcomes 2012 (36).

In our study, VAI were designed as an exposure variable, albuminuria, low-eGFR and CKD were treated as outcome variables.

### 2.4. Selection of covariates

Several potential covariates that may confound the association between VAI and decreased renal function were summarized in our analysis, including gender, age, race, education level, smoking status, BMI, serum creatinine, serum uric acid, total cholesterol, ALT, AST, hypertension and diabetes. According to BMI, participants were considered normal weight, overweight and obese when their BMI fell between <25, 25–29.9, and ≥30 kg/m^2^. In addition, we also treated gender (male/female), age (<60/≥60 years), BMI (normal weight/overweight/obesity), hypertension (yes/no), diabetes (yes/no) as stratified factors to conduct subgroup analysis and pre-specified effect modifiers to evaluate the interaction effect. All details regarding these variables are available on the website at www.cdc.gov/nchs/nhanes/.

### 2.5. Statistical analysis

According to NHANES analytic guidelines, statistical analyses were performed with appropriate sampling weights and accounting for complex multistage cluster surveys. Means with standard error (SE) was calculated for continuous variables, and proportions were calculated for categorical variables. Participants grouped by VAI tertiles were compared using a weighted Student’s *t*-test (for continuous variables) or a weighted chi-square test (for categorical variables). Three different models were analyzed using multivariable logistic regression to determine the effects of VAI on outcome variables (albuminuria, low eGFR, and CKD). In model 1, no covariates were adjusted. Model 2 was adjusted for gender, age and race. Model 3 was adjusted for gender, age, race, education level, BMI, ALT, AST, serum creatinine, serum uric acid, total cholesterol, hypertension, diabetes and smoking status. To reduce the potential bias and enhance the reliability of our study, first, we conducted all analysis with the consideration of NHANES sampling weights to make our samples more representative and reduce the selection bias. In addition, we adjusted for many confounding covariates to reduce the confounding bias. We also treated VAI as tertiles to evaluate the robustness in sensitivity analysis. Subgroup analyses of the associations of VAI with albuminuria, low-eGFR and CKD were conducted with stratified factors, including gender (male/female), age (<60/≥60 years), BMI (normal weight/overweight/obesity), hypertension (yes/no), and diabetes (yes/no). In addition, the stratified factors were also treated as pre-specified potential modifiers, with an interaction term added to measure heterogeneity among subgroups. Input of missing values was done by median for continuous variables or mode for categorical variables. All analyses were preformed using R version 3.4.3 (The R Foundation)^1^ and Empower software (X&Y Solutions, Inc., Boston MA, USA).^2^ The level of statistical significance was set at *P* < 0.05.

## 3. Results

### 3.1. Participants characteristics at baseline

There were 35,018 participants enrolled in this study, whose average age was 46.44 ± 0.23 years. Among them, 49.20% were male and 50.80% were female. The prevalence rates of albuminuria, low-eGFR and CKD was 5.18, 6.42, and 10.62%, respectively. Participants in higher VAI tertiles had increased rates of albuminuria, low-eGFR, CKD as well. In the lowest VAI tertile participants, 3.93% had albuminuria, 4.23% had low-eGFR and 7.65% had CKD. In the middle VAI tertile participants, 5.17% had albuminuria, 6.26% had low-eGFR and 10.54% had CKD. Participants in the highest VAI tertile showed the highest rates of albuminuria (6.44%), low-eGFR (8.78%) and CKD (13.67%). Age, gender, smoking status, BMI, diabetes, hypertension, serum creatinine, serum uric acid, total cholesterol, HDL-C, ALT, AST, waist circumference, TGs, urinary albumin and ACR were significantly different among the VAI tertiles (all *P* < 0.05). Compared to the lowest VAI group, participants with increased VAI group were significantly more likely to have hypertension, diabetes, elevated BMI, serum uric acid, total cholesterol, ALT, AST, waist circumference, TGs, urinary albumin, ACR and decreased HDL-C levels (all *P* < 0.05). There was no statistically significant difference between tertiles in race, education level or urinary creatinine (all *P* > 0.05) (Table 1).

**TABLE 1:** Baseline characteristics according to visceral adiposity index tertiles.

VAI	Overall	Tertile 1	Tertile 2	Tertile 3	P for trend
Age (year)	46.44 ± 0.23	43.35 ± 0.32	46.87 ± 0.24	49.09 ± 0.27	<0.0001
**Gender, % (SE)**					
Male	49.20 (0.27)	49.33 (0.54)	46.90 (0.62)	51.40 (0.62)	0.0308
Female	50.80 (0.27)	50.67 (0.54)	53.10 (0.62)	48.60 (0.62)	
**Race, % (SE)**					
Mexican American	8.76 (0.68)	6.48 (0.51)	9.05 (0.73)	10.76 (0.90)	0.6185
Other Hispanic	5.63 (0.44)	4.90 (0.43)	5.71 (0.45)	6.27 (0.53)	
Non-Hispanic white	67.29 (1.27)	65.33 (1.25)	67.36 (1.37)	69.19 (1.43)	
Non-Hispanic black	10.68 (0.66)	15.71 (0.93)	10.35 (0.64)	5.98 (0.45)	
Other Races	7.64 (0.38)	7.58 (0.43)	7.53 (0.46)	7.80 (0.48)	
**Education level, % (SE)**					
Less than high school	16.28 (0.55)	13.24 (0.53)	16.22 (0.70)	19.38 (0.69)	0.1927
High school or GED	23.61 (0.47)	21.64 (0.68)	23.21 (0.59)	25.98 (0.68)	
Above high school	60.06 (0.81)	65.10 (0.92)	60.52 (0.92)	54.56 (0.92)	
Others	0.05 (0.01)	0.03 (0.01)	0.05 (0.02)	0.07 (0.03)	
**Smoking status, % (SE)**					
Never	55.37 (0.55)	59.59 (0.79)	55.90 (0.71)	50.71 (0.70)	<0.0001
Former	24.44 (0.41)	22.31 (0.59)	24.34 (0.60)	26.64 (0.64)	
Current	20.18 (0.44)	18.10 (0.55)	19.76 (0.61)	22.65 (0.60)	
BMI (Kg/m^2^)	28.84 ± 0.08	25.94 ± 0.09	29.12 ± 0.11	31.45 ± 0.09	<0.0001
Diabetes, % (SE)	8.95 (0.22)	4.44 (0.23)	7.94 (0.32)	14.50 (0.47)	<0.0001
Hypertension, % (SE)	30.54 (0.47)	20.99 (0.57)	30.33 (0.65)	40.32 (0.68)	<0.0001
SCr (μmol/L)	78.21 ± 0.21	77.13 ± 0.33	77.95 ± 0.30	79.55 ± 0.32	<0.0001
Serum uric acid (μmol/L)	322.21 ± 0.74	300.57 ± 0.98	321.35 ± 1.16	344.75 ± 1.22	<0.0001
TC (mmol/L)	5.01 ± 0.01	4.76 ± 0.02	4.97 ± 0.02	5.30 ± 0.02	<0.0001
HDL-C (mmol/L)	1.38 ± 0.01	1.70 ± 0.01	1.36 ± 0.00	1.08 ± 0.00	<0.0001
ALT (U/L)	25.27 ± 0.14	22.26 ± 0.22	24.56 ± 0.17	29.00 ± 0.26	<0.0001
AST (U/L)	25.24 ± 0.11	24.96 ± 0.20	24.45 ± 0.15	26.32 ± 0.20	<0.0001
Triglycerides (mmol/L)	1.71 ± 0.01	0.79 ± 0.00	1.38 ± 0.00	2.98 ± 0.02	<0.0001
Waist circumference (cm)	98.69 ± 0.22	90.29 ± 0.23	99.34 ± 0.25	106.46 ± 0.23	<0.0001
Albumin, urine (mg/L)	32.26 ± 1.26	23.05 ± 1.29	26.35 ± 1.50	47.46 ± 3.47	<0.0001
Creatinine, urine (mg/dL)	122.32 ± 0.83	121.67 ± 1.39	121.80 ± 1.11	123.49 ± 1.05	0.2675
ACR (mg/g)	31.30 ± 1.27	20.83 ± 1.23	26.55 ± 1.76	46.60 ± 3.36	<0.0001
eGFR (mL/min/1.73 m^2^)	94.82 ± 0.31	98.53 ± 0.38	94.32 ± 0.36	91.60 ± 0.36	<0.0001
Albuminuria, % (SE)	5.18 (0.17)	3.93 (0.21)	5.17 (0.26)	6.44 (0.37)	<0.0001
Low-eGFR, % (SE)	6.42 (0.22)	4.23 (0.28)	6.26 (0.28)	8.78 (0.38)	<0.0001
CKD, % (SE)	10.62 (0.26)	7.65 (0.33)	10.54 (0.37)	13.67 (0.50)	<0.0001

GED, general educational development; BMI, body mass index; SCr, serum creatinine; TC, total cholesterol; HDL-C, high density lipoprotein-cholesterol; ALT, alanine transaminase; AST, aspartate transaminase; ACR, albumin: creatinine ratio; eGFR, estimated-glomerular filtration rate; CKD, chronic kidney disease.

### 3.2. A positive association of visceral adiposity index and albuminuria

Our results showed that a higher VAI was associated with an increased risk of albuminuria. This association was significant both in our crude model (OR = 1.04, 95% CI: 1.03, 1.06) and minimally adjusted model (OR = 1.06, 95% CI: 1.04, 1.09). After full adjustment, a positive association between VAI and albuminuria still remained stable (OR = 1.03, 95% CI: 1.00, 1.06), indicating that each unit of VAI score was associated with a 4% increase in albuminuria risk. When treating VAI as tertiles, participants in the highest VAI tertile had a significantly 30% increased risk of albuminuria compared with those in the lowest tertile (OR = 1.30, 95% CI: 1.07, 1.58) (Table 2).

**TABLE 2:** Association between visceral adiposity index and decreased renal function.

Visceral adiposity index group	Albuminuria OR^1^ (95% CI^2^), *P*-value	Low-eGFR OR (95% CI), *P*-value	CKD OR (95% CI), *P*-value
**Crude model (Model 1)^3^**			
Continuous	1.04 (1.03, 1.06), <0.0001	1.04 (1.03, 1.05), <0.0001	1.04 (1.03, 1.06), <0.0001
**Categories**			
Tertile 1	Reference	Reference	Reference
Tertile 2	1.33 (1.16, 1.52), 0.0001	1.51 (1.31, 1.75), <0.0001	1.42 (1.27, 1.59), <0.0001
Tertile 3	1.68 (1.43, 1.98), <0.0001	2.18 (1.88, 2.53), <0.0001	1.91 (1.69, 2.16), <0.0001
P for trend	<0.0001	<0.0001	<0.0001
**Minimally adjusted model (Model 2)^4^**			
Continuous	1.06 (1.04, 1.09), <0.0001	1.06 (1.04, 1.08), <0.0001	1.05 (1.03, 1.07), < 0.0001
**Categories**			
Tertile 1	Reference	Reference	Reference
Tertile 2	1.20 (1.04, 1.38), 0.0142	1.30 (1.10, 1.54), 0.0028	1.19 (1.05, 1.35), 0.0087
Tertile 3	1.61 (1.35, 1.92), <0.0001	2.01 (1.70, 2.37), <0.0001	1.59 (1.39, 1.83), <0.0001
P for trend	<0.0001	<0.0001	<0.0001
**Fully adjusted model (Model 3)^5^**			
Continuous	1.03 (1.00, 1.06), 0.0336	1.00 (0.93, 1.08), 0.9325	1.04 (1.02, 1.06), 0.0005
**Categories**			
Tertile 1	Reference	Reference	Reference
Tertile 2	1.14 (0.98, 1.34), 0.0952	1.65 (0.82, 3.31), 0.1633	1.09 (0.95, 1.25), 0.2474
Tertile 3	1.30 (1.07, 1.58), 0.0108	1.24 (0.56, 2.72), 0.5932	1.27 (1.08, 1.49), 0.0056
P for trend	0.0118	0.8004	0.0050

In sensitivity analysis, visceral adiposity index was converted from a continuous variable to a categorical variable (tertiles).

^1^95% CI: 95% confidence interval.

^2^OR: odd ratio.

^3^Model 1: no covariates were adjusted.

^4^Model 2: adjusted for gender, age, and race.

^5^Model 3: adjusted for gender, age, race, education level, body mass index, ALT, AST, serum creatinine, serum uric acid, total cholesterol, hypertension, diabetes, and smoking status.

### 3.3. Visceral adiposity index and low-eGFR

We also estimated the association of VAI with low-eGFR in three different models. We found a significant positive association between VAI and low-eGFR both in both the crude (Model 1: OR = 1.04, 95% CI: 1.03, 1.05) and minimally adjusted models (Model 2: OR = 1.06, 95% CI: 1.04, 1.08). Despite this, this positive relationship did not reach statistical significance in fully adjusted analysis (OR = 1.00, 95% CI: 0.93, 1.08). Additionally, they did not show any statistically significant association when VAI was treated as tertiles (Table 2).

### 3.4. A positive association of visceral adiposity index and chronic kidney disease

For CKD, we also found a positive association between VAI and the increased likelihood of CKD with statistical significance. In our crude model and minimally adjusted model, participants with a higher VAI tended to show an increased risk of CKD (Model 1: OR = 1.04, 95% CI: 1.03, 1.06; Model 2: OR = 1.05, 95% CI: 1.03, 1.07). A unit higher of VAI increased CKD risk by 4% after full adjustment (Model 3: OR = 1.04, 95% CI: 1.02, 1.06). Even after treating VAI as tertiles, there was still a statistically significant association. A significant 27% higher risk was experienced by participants in the highest VAI tertile compared to those in the lowest VAI tertile (OR = 1.27, 95% CI: 1.08, 1.49) (Table 2).

### 3.5. Subgroup analysis

**FIGURE 2:**
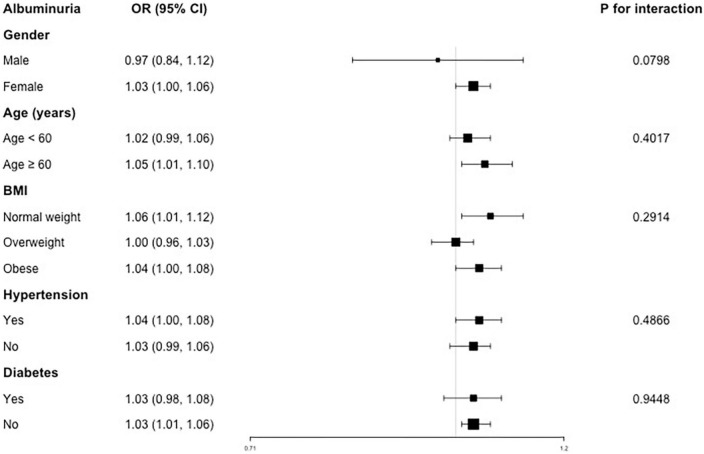
Subgroup analysis for the association between VAI and albuminuria.

Our results indicated that the associations of VAI level with decreased renal function were not consistent. A significant relationship of VAI with albuminuria was detected in female (OR = 1.03), age ≥60 years (OR = 1.05), normal weight (OR = 1.06) and obese (OR = 1.04), hypertension (OR = 1.04) and non-diabetes subjects (OR = 1.03), respectively. In the interaction test, VAI and albuminuria were not significantly associated with each stratification (Figure 2).

**FIGURE 3:**
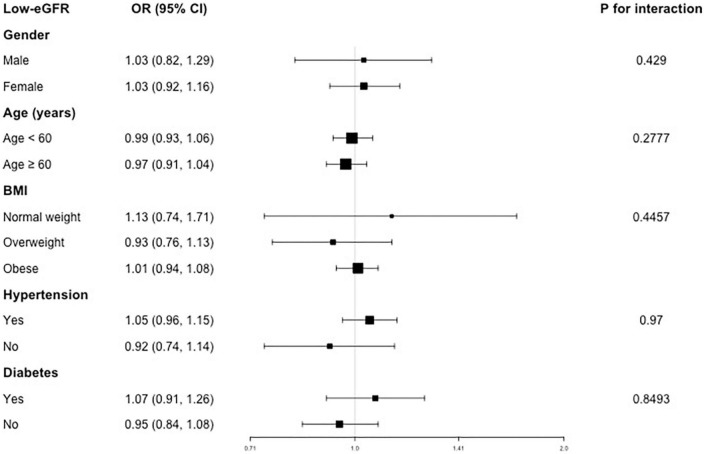
Subgroup analysis for the association between VAI and low-eGFR.

For the association between VAI and low-eGFR, consistent previous results, we still did not observe any statistically significant relationship (Figure 3).

**FIGURE 4:**
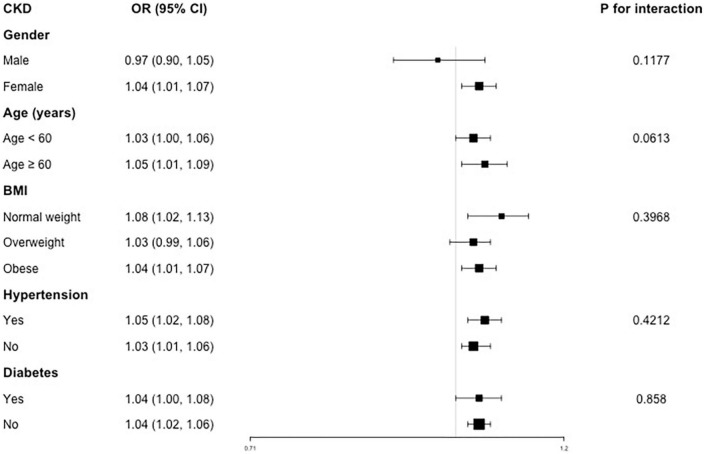
Subgroup analysis for the association between VAI and CKD.

For CKD, a positive association was found in females (OR = 1.04), both age <60 (OR = 1.03) and ≥60 years (OR = 1.05), normal weight (OR = 1.08) and obese (OR = 1.04), both hypertension (OR = 1.05) and non-hypertension (OR = 1.03), both diabetes (OR = 1.04) and non-diabetes (OR = 1.04) subjects (Figure 4). In addition, there was no significant difference suggested by the interaction test among different stratifications, indicating that this positive association was not significantly influenced by gender, age, BMI, hypertension and diabetes on this positive association (all P for interaction >0.05).

## 4. Discussion

Our cross-sectional study with 35,018 participants found that participants with higher VAI were more likely to have albuminuria and CKD. Subgroup analysis and interaction tests revealed similar associations across different populations. Based on our results, visceral adiposity accumulation should be considered, and the management of visceral fat distribution may alleviate the decrease in renal function. Our results also indicated the negative effects of the accumulation of visceral adiposity on renal health. Since the calculation of VAI was simple and cheap, and it could distinguish visceral adiposity from subcutaneous adiposity accurately, individuals should pay more attention to this mathematical parameter for their health.

Our study assessed the association between VAI and decreased renal function. The impact of the VAI on renal function has been previously evaluated in the different regions and populations (37–43). A study conducted in 6,693 non-diabetic participants in Iran observed that VAI seems to be an independent predictor of renal function decline only in males (37). A cross-sectional study that enrolled 4,947 participants in Korea confirmed that the VAI was a good predictor of the pathogenesis of CKD in men but not in women (38). However, Dai and colleagues has previously demonstrated that VAI was significantly associated with CKD in women in rural population of northeast China (39). A recent study of 400 individuals aged 50–90 years also reported a stronger correlation of VAI with CKD in Taiwan, China, especially for middle-aged and elderly females (40). Similarly, Huang et al. reported a consistent result based on a cross-sectional study that included 2,142 individuals in South China (41). Furthermore, a population-based study of 15,159 participants conducted by Bamba et al. demonstrated that VAI can be a predictor of incident CKD in both males and females in Japan (42). Wen et al. also showed a clear association between the VAI and urinary albumin excretion in Chinese type 2 diabetic patients regardless of the gender (43). Similarly, we highlighted the negative effects of visceral adiposity accumulation on renal health and our present study was consistent with those reports of Bamba et al. and Wen et al., suggesting that higher VAI indicated increased likelihood of albuminuria and CKD. As for different results reported before, we think the variance of population characteristics, including race, region, sample size, CKD definition, and eGFR-related calculation methods may contribute to the discrepancy among these studies. In addition, the results without adjustment for established risk factors for CKD, such as blood pressure, plasma glucose, and serum low density lipoprotein-cholesterol, might lead to misleading conclusions as well (44). VAI can distinguish visceral adiposity from subcutaneous adiposity accurately compared to some other body assessment parameters, such as BMI, WC, and WHtR. For the application of VAI in clinical practice, we think individuals can measure the VAI index of each patient and stratify patients’ risk according to the VAI. According to different risk stratification of patients, more targeted health management for patients could be conducted. In addition, considering the negative effect of visceral fat on renal health, individuals can take the initiative to change their lifestyle, use drugs and other methods to reduce their visceral obesity.

The association between obesity and an increased risk of incident CKD (45, 46), end-stage renal disease (47, 48), and mortality (49, 50) has already been demonstrated by previous studies. In addition to VAI, other visceral adiposity accumulation indicators, such as lipid accumulation product (LAP), BMI, WC, WHtR, and waist-to-hip ratio (WHR), also have clinical predictive effects. BMI is the most classic indicator in the assessment of adiposity (37). A meta-analysis including 39 cohorts covering 630,677 participants revealed that higher BMI was associated with an increased risk of low-eGFR (hazard ratio, HR = 1.02, 95% CI: 1.01–1.03) and albuminuria (HR = 1.02, 95% CI: 1.00–1.04) (51). However, due to the deficiency in distinguishing between fat and muscle as well as between subcutaneous and visceral fat tissues, BMI may lead to bias in measuring the effects of obesity on health outcomes (52). In a prospective study of a Korean population, Oh H et al. reported that WC, not BMI, could predict a decline in renal function. Simultaneously, WHR and WHtR were reported to be associated with renal function decline as well (53). Moreover, Elsayed et al. revealed that the assessment of CKD risk should use WHR rather than BMI as an anthropomorphic measure of obesity (54). Unfortunately, both WC and WHR have limited accuracy in distinguishing between visceral adipose tissue (VAT) and subcutaneous adipose tissue (SAT) (55). Previous studies have shown that LAP and VAI are superior to BMI, WC, WHtR and WHR in the evaluation of renal function decline in clinical practice (37, 39). Furthermore, Mousapour et al. suggested that while LAP and VAI outperform BMI, WC, WHtR and WHR, VAI could be an independent predictor of renal function decline in non-diabetic males (37). Similarly, our study focused on the association between VAI and decreased renal function, and detected a positive relationship of higher VAI with increased likelihood of albuminuria and CKD. Since VAI was a reliable parameter of visceral fat, our results highlighted the negative effects of visceral obesity on renal health.

Several potential mechanisms may explain the association of VAI and decreased kidney function. Visceral adiposity has a positive relationship with the development of inflammation, oxidative stress, endothelial dysfunction, and atherosclerosis, resulting in glomerulosclerosis and tubulointerstitial fibrosis (56–58). Thus, it may lead to a decrease in kidney function. Adiposity accumulation could induce pro-inflammatory pathways, including interleukin-6 (IL-6), tumor necrosis factor-alpha (TNF-α), and transforming growth factor-beta (TGF-β), as well as augment the production of reactive oxygen species (59, 60). Furthermore, this accumulation could also activate the renin-angiotensin-aldosterone system (RAAS), causing hypertension and increasing insulin resistance, which are recognized renal injury factors (61, 62). Additionally, the distribution of central fat might increase the glomerular filtration rate relative to effective renal plasma flow, leading to an increased filtration fraction, and ultimately to glomerular hyper-perfusion, hypertension and even functional loss (63). In addition, it was noting that participants in the third tertile of VAI seems to have worse conditions, such as higher age, obesity, hypertension, diabetes and disorder of lipid metabolism, these comorbidities may contribute to the increased risk of decreased renal function.

Although we detected a significantly positive association of VAI and CKD, it may be mediated by some other diseases and conditions. Using same NHANES dataset, Ciardullo et al. found that participant with MS (both obese and non-obese) showed higher prevalence of albuminuria and reduced eGFR compared with those without obesity or MS, while there was no significant difference in those with MS but without obese. They suggested that MS and but not increased body fat alone was related with CKD (64). Indeed, the association between MS and CKD was controversial. Some studies did not find increased risk of CKD in metabolically healthy obese individuals compared to metabolically healthy non-obese individuals, while others found a residual increase in CKD risk that remained (65–69). A possible explanation of these inconsistencies is related to different definitions of CKD and MS. Some studies did not include albuminuria as an indicator of CKD and the MS diagnostic criteria varied among different studies as well. In addition to MS, NAFLD and related fibrosis may also influence the relationship between VAI and CKD. The correlation of VAI with NAFLD has been reported before (23, 24). Meanwhile, liver fibrosis, but not steatosis, has been proven to associated with reduced kidney function independently (70). In a meta-analysis enrolled seven cross-sectional studies, Ciardullo et al. reported that elevated liver stiffness was associated with higher likelihood of kidney outcomes including albuminuria and CKD among patients with NAFLD (71). Taken together, at least part of the association between VAI and CKD may be mediated by MS, NAFLD and associated fibrosis, etc. In our analysis, VAI is calculated considering TG and HDL levels, which are MS parameters. Even VAI representing visceral fat accumulation, perhaps some metabolism-related factor could have influenced our results as well. Thus, more large-scale prospective studies are still needed to valid our findings.

This study has several strengths. First, the sample selection and sample size are representative and sufficient. To our knowledge, the present study included the largest number of samples on this topic. Additionally, we adjusted for confounding covariates to reduce the confounding bias. Therefore, more reliable conclusions can be obtained. However, the limitations should also be noted. Our cross-sectional study design did not permit us to establish a causal relationship. Prospective studies with larger sample sizes are needed to clarify this issue. In addition, even though some potential covariates have been adjusted, other potentially confounding variables could not be completely excluded, such as the use of drugs including diuretics and steroids, etc. The use of a single spot of urine to evaluate albuminuria is a limitation as well. Although it is a validated method, is not the best option compared to 24 h urine.

## 5. Conclusion

Elevated VAI levels were independently associated with albuminuria and CKD, which highlights the importance of managing decreased renal function in patients with visceral adipose accumulation. However, the validity of our findings needs to be further confirmed by large-scale prospective studies.

## Data availability statement

Publicly available datasets were analyzed in this study. This data can be found here: https://www.cdc.gov/nchs/nhanes/.

## Ethics statement

The studies involving human participants were reviewed and approved by the National Center for Health Statistics (NCHS) Research Ethics Review Board. The patients/participants provided their written informed consent to participate in this study.

## Author contributions

ZQ: software, data analysis, and writing—original draft. XC: writing—original draft, formal analysis, and methodology. JS: data analysis. LJ: conceptualization, funding acquisition, and writing—reviewing and editing. All authors contributed to the article and approved the submitted version.

## Footnotes

1
http://www.R-project.org

2
www.empowerstats.com
